# Association of Hyperautofluorescence Signals with Geographic Atrophy Progression in the METformin for the MINimization of Geographic Atrophy Progression Trial

**DOI:** 10.1016/j.xops.2024.100620

**Published:** 2024-09-12

**Authors:** Abu Tahir Taha, Liangbo Linus Shen, Antonio Diaz, Noor Chahal, Jasmeet Saroya, Mengyuan Sun, Michael J. Allingham, Sina Farsiu, Glenn Yiu, Jeremy D. Keenan, Jay M. Stewart

**Affiliations:** 1 Department of Ophthalmology, University of California, San Francisco, San Francisco, California; 2 Institute of Cardiovascular Diseases, Gladstone Institute, San Francisco, California; 3 Department of Ophthalmology, Duke University Medical Center, Durham, North California; 4 Department of Ophthalmology & Visual Sciences, University of California, Davis, Sacramento, California; 5 University of California, San Francisco, Francis I Proctor Foundation, San Francisco, California; 6 Department of Ophthalmology, Zuckerberg San Francisco General Hospital and Trauma Center, San Francisco, California

**Keywords:** AMD, Geographic atrophy, RAFH

## Abstract

**Purpose:**

To investigate the association between rim area focal hyperautofluorescence (RAFH) signals and geographic atrophy (GA) growth rates, as well as the impact of oral metformin on the longitudinal change of RAFH.

**Design:**

Secondary analysis of a randomized controlled trial.

**Participants:**

Seventy-one eyes from 44 participants with GA and ≥6 months of follow-up in the METformin for the MINimization of geographic atrophy progression study.

**Methods:**

Fundus autofluorescence images were captured using a 488 nm excitation wavelength. Two masked graders identified and measured RAFH lesions using proprietary semiautomatic segmentation software and ImageJ. We calculated RAFH by dividing the areas of hyperautofluorescence within a 450-μm rim circumscribing the GA by the total area enclosed within this rim.

**Main Outcome Measures:**

Longitudinal changes in RAFH and GA area.

**Results:**

Baseline RAFH was positively associated with the baseline square root of GA area 0.065/year (*P* < 0.001). In the entire study cohort, higher baseline RAFH was associated with a faster GA area growth rate in mm^2^/year (Spearman’s ρ = 0.53; *P* < 0.001). The association became weaker in square root-transformed GA area growth (ρ = 0.19, *P* = 0.11) and perimeter-adjusted GA growth rate (ρ = 0.28, *P* = 0.02), achieving statistical significance only in the latter. When this analysis was stratified into 3 baseline GA tertiles, the first and second tertiles showed weak to moderate association with statistical significance in all 3 modes of GA growth rates. Rim area focal hyperautofluorescence increased slightly but significantly over time at 0.020/year (*P* < 0.01). Rim area focal hyperautofluorescence increased slightly but significantly over time at 0.020/year (*P* < 0.01). The use of oral metformin was not significantly associated with the change in RAFH over time compared with the observation group (0.023/year vs. 0.016/year; *P* = 0.29).

**Conclusions:**

Increased baseline RAFH is associated with faster GA area progression. However, the effect size of this association may depend on the baseline GA lesion size such that small to medium-sized GA lesions display this relationship regardless of the mode of the calculation of GA growth rate.

**Financial Disclosures:**

Proprietary or commercial disclosure may be found in the Footnotes and Disclosures at the end of this article.

Geographic atrophy (GA), an advanced form of age-related macular degeneration, affects the vision of >5 million individuals globally.^1^ Perifoveal atrophy impacts visual functions such as reading, driving, and seeing in low-light conditions. In contrast, foveal involvement can significantly impair central visual acuity. Geographic atrophy is characterized by the progressive deterioration of photoreceptors, retinal pigment epithelium (RPE), Bruch’s membrane, and the choriocapillaris, primarily in the macula.^2^ The United States Food and Drug Administration recently approved 2 complement-factor inhibitors for slowing GA progression, and clinical trials evaluating other therapies are underway.^3^, ^4^, ^5^, ^6^, ^7^

The enlargement rate or the enlargement of GA area over time assessed by fundus autofluorescence (FAF) imaging is the most common primary endpoint in GA clinical trials.^3^^,^^4^^,^^8^ However, the GA growth rate varies widely across different patients.^8^, ^9^, ^10^, ^11^, ^12^ Several studies have investigated various biomarkers on FAF imaging to stratify the progression rate of GA.^8^^,^^13^, ^14^, ^15^, ^16^ One such biomarker is hyperautofluorescent signals in the junctional zone of existing GA lesions. Prior studies suggest that hyperautofluorescence patterns in the junctional zone may be associated with GA enlargement rates.^17^, ^18^, ^19^, ^20^ However, grading these patterns is subjective, which poses a challenge in clinical trials that use these patterns as inclusion or exclusion criteria.^21^, ^22^, ^23^ Alternatively, a few studies have attempted to quantify junctional hyperautofluorescent signals to establish their associations with GA growth.^24^, ^25^, ^26^ Bearelly et al defined rim area focal hyperautofluorescence (RAFH) as the percentage of area with increased autofluorescence within the 500-μm border around GA.^25^ This concept was further improved by Allingham et al using semiautomatic software that quantified RAFH as a ratio of hyperautofluorescent areas over the total amount of area enclosed within a 450-μm border around GA (Fig 1).^24^ These studies found that the enlargement rate of GA area (in mm^2^/year) was positively associated with the baseline RAFH. However, these prior studies did not account for baseline GA lesion size or peri GA area.^27^, ^28^, ^29^, ^30^ Thus, the nature of the relationship between junctional hyperautofluorescent signals and GA growth rate remains unclear. Additionally, junctional hyperautofluorescent regions may correspond with disease activity,^26^^,^^27^^,^^31^ but to our knowledge, the longitudinal change of RAFH remains understudied.

**Figure 1:**
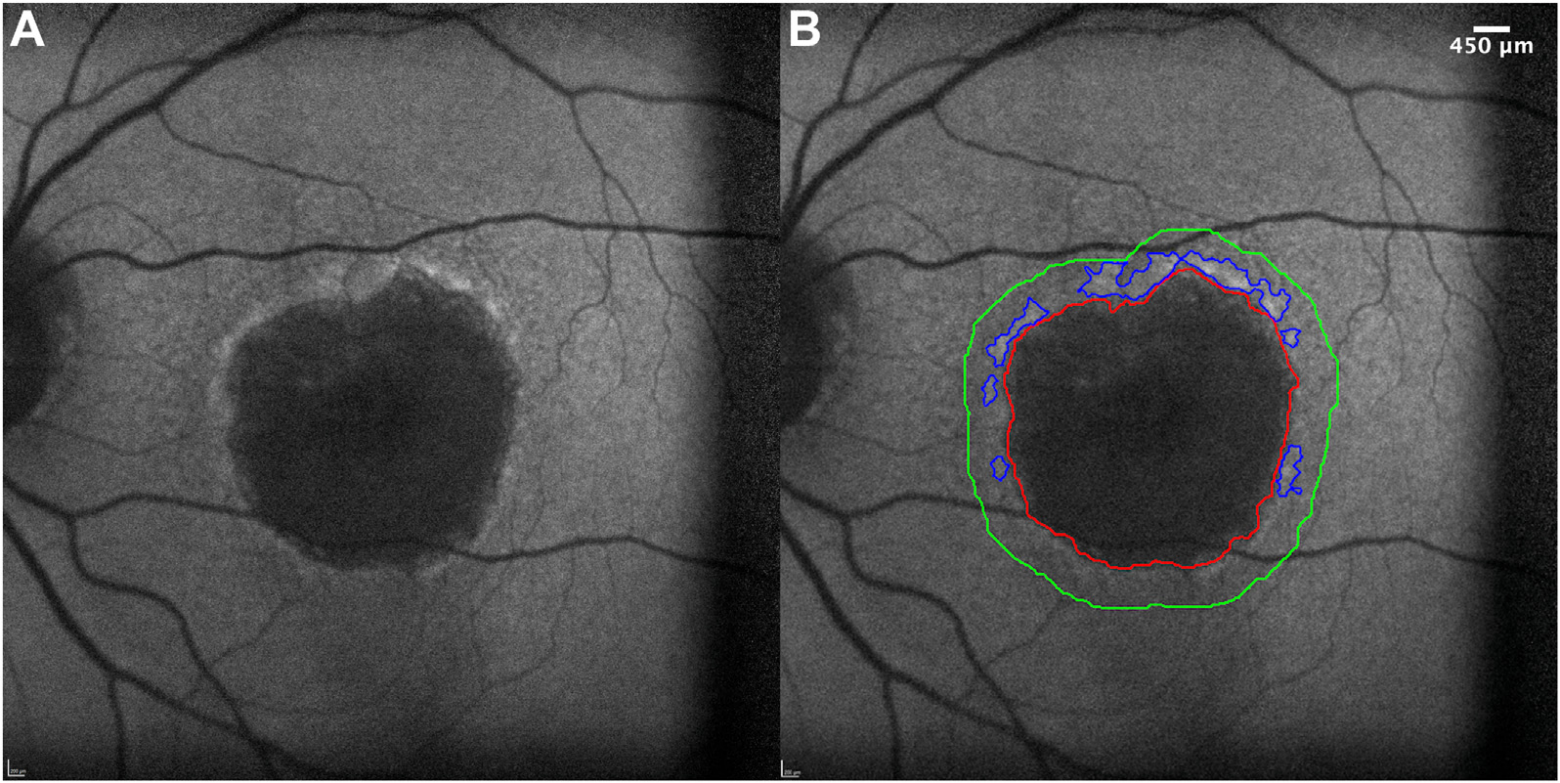
Blue autofluorescence image of the left eye of a 79-year-old female with a past medical history of smoking and cardiovascular disease without a history of ocular interventions who was diagnosed with age-related macular degeneration in 2000. This participant was enrolled in the study in 2017 with a best-corrected visual acuity of 49 letters. **A,** Shows the raw image autofluorescence image before any processing. **B,** Depicts the same image with superimposed delineation of GA in red, junctional hyperautofluorescent areas in blue, and the 450-μm border in green. GA grading was adopted from the METformin for the MINimization of Geographic Atrophy Progression trial and the shown hyperautofluorescence areas were delineated by the software. After correcting any perceived errors in the identified hyperautofluorescent areas, rim area focal hyperautofluorescence was calculated using a custom-made code in MATLAB by dividing the total area in blue by the area interposed between the 450-μm border and the GA lesion. GA = geographic atrophy.

Building on prior work, we investigated the association of baseline RAFH with the GA growth rate while adjusting for baseline GA lesion size. We further explored whether baseline RAFH is associated with other clinical and demographic factors known to be associated with the progression of GA.^32^^,^^33^ We also used data from the METformin for the MINimization of Geographic Atrophy Progression (METforMIN) trial, which did not find a benefit to oral metformin in slowing down GA progression, to investigate the change in RAFH over time and to determine whether oral metformin impacts its overall growth.^34^

## Methods

### Study Design and Eligibility Criteria

The current study is a secondary analysis of METforMIN clinical trial (ClinicalTrials.gov identifier: NCT02684578).^34^ METformin for the MINimization of Geographic Atrophy Progression was a multicenter phase II clinical trial that investigated the efficacy of oral metformin in slowing down the rate of progression of GA. The study protocol, design, and primary results of the clinical trial design can be found in our previous publication.^34^ Briefly, we recruited nondiabetic participants >55 years of age with GA secondary to nonexudative age-related macular degeneration from 12 clinical sites. These patients were selected based on the investigator’s expertise at each clinical site. The items considered during this assessment included the history of the disease, clinical examination, and imaging findings. We randomized 66 eligible participants in a 1:1 ratio to either metformin or observation. Participants in the metformin arm were instructed to gradually increase the metformin dose to 1000 mg twice daily, which participants took for 18 months. These participants were then followed for an additional 6 months without treatment. In the observation arm, we followed participants according to the standard of care every 6 months for 24 months. We obtained FAF and OCT imaging using Heidelberg Spectralis with BluePeak FAF (488 nm excitation wavelength). Our current analysis included 71 eyes (34 metformin; 37 observation) from 44 participants (21 metformin; 23 observation) with GA area measurements at the baseline and ≥1 follow-up visit. We investigated the growth of RAFH over 24 months and the impact of metformin on RAFH change for 18 months. In a separate supplementary analysis applicable only to the aim of investigating the impact of metformin on RAFH over 18 months, we excluded participants who were not adherent to taking metformin ≥75% of the time (3 participants). We included 66 eyes (29 metformin; 37 observation) from 41 (18 metformin; 23 observation) participants in the analysis.

The study was conducted in accordance with the tenets of the Declaration of Helsinki and was approved by the institutional review board at each clinical site. The METforMIN trial obtained written informed consent at enrollment and complied with the Health Insurance Portability and Accountability Act.

### Outcome Measures and RAFH Grading

We performed a comprehensive eye examination during each visit, obtained FAF and OCT imaging, and assessed participants’ adherence to metformin. We also obtained other clinical and demographic information such as sex, race/ethnicity, body mass index (BMI), and history of smoking or cardiovascular disease (CVD).

We used information from the METforMIN trial to obtain the following FAF image characteristics:^34^ area of GA lesions, baseline focality of GA lesions (unifocal vs. multifocal), and classification of junctional hyperautofluorescent patterns (group 1: “None” and “Focal”; group 2: “Banded,” “Patchy,” and “Diffuse”).^18^^,^^35^ A simpler FAF hyperautofluorescent classification was chosen to improve intergrader reproducibility.^36^ Specifically, 2 independent graders, who were masked to the treatment allocation of the patients, graded each FAF image for the total GA area and specified the FAF phenotype. Two expert graders further reviewed GA gradings to ensure accuracy. A detailed description of GA grading and FAF phenotyping protocol can be found in the Supplemental Methods of this manuscript, available at https://www.ophthalmologyscience.org.

For the present study, 2 masked graders (A.T.T. and A.D.) used a validated proprietary semiautomatic software^24^ to identify the perilesional 450-μm border and potential hyperautofluorescent regions of interest. A detailed description of the hyperautofluorescent signals thresholding process can be read in the original publication by Allingham et al.^24^ Briefly, once the software drew the perimeter surrounding the GA lesions, it applied a local threshold of +40 pixel value (with intensity values ranging from 0 to 255; 0 represents black, and 255 represents white) to the circumscribed region between the perimeter and the GA. It then marked areas above this threshold as hyperautofluorescent. Both graders independently corrected any perceived tracing errors and then delineated hyperautofluorescent regions within the 450-μm border in the FAF images using ImageJ (National Institutes of Health).^37^ The 450-μm width was chosen based on the prior reports, which noted most hyperautofluorescent areas to be confined within a width of approximately 450 μm.^24^^,^^25^^,^^38^ An example delineation of RAFH as performed by the software before any manual alterations can be seen in Figure 1B. We calculated RAFH as the sum of all hyperautofluorescent areas within the 450-μm border divided by the total area enclosed between GA and its 450-μm border using a custom code in MATLAB (MathWorks).^39^ Each grader independently graded the entire image set. For images with RAFH outside the 95% limits of agreement between the 2 graders, both graders assessed their gradings together, discussed their approach, and reached mutual consensus.

### Statistical Analysis

We used R software^40^ (version 4.0.4, R Foundation for Statistical Computing) to perform all statistical analyses. We used a Bland–Altman plot and intraclass correlation coefficients to assess the reliability between the 2 graders. We calculated the mean RAFH measurements between the 2 graders for all subsequent analyses. We used Spearman’s rank correlation (ρ)^24^, ^25^, ^26^ to determine the association between the baseline RAFH and growth rate of GA area (mm^2^/year), the square root-transformed GA growth rate (mm/year), and perimeter-adjusted GA growth rate (mm/year). We calculated the annual GA growth rate (mm^2^/year) by subtracting the GA area at the first visit (mm^2^) from the GA area at the last visit (mm^2^) and dividing the result by the time interval (years). We included GA growth rate to compare our results with previous studies reporting on GA progression, which also utilize this method of quantifying GA growth. Furthermore, recent GA clinical trials have also report GA growth in mm^2^.^3^^,^^4^ We calculated the square root-transformation of GA growth rate (mm/year) by subtracting the square root of GA at the first visit (mm) from that at the last visit (mm) and divided the result by the time interval (years). Lastly, we determined perimeter-adjusted GA growth rate by dividing the GA area growth (mm^2^/year) by the mean GA perimeter between the first and the last visits (mm).^30^ We further stratified the list of baseline GA areas of the 71 eyes into 3 tertiles, splitting them into 3 equal groups based on the size of the GA area.^41^ The tertiles-based method to account for the potential impact of baseline GA area on the subsequent growth rate has been replicated previously by other studies.^29^^,^^42^^,^^43^ We then repeated the same analysis to establish the association between RAFH and GA growth rate within each tertile. We used a univariate linear mixed-effects regression model with a random effect for participant to account for intereye correlations (“lme4” R package)^44^ to investigate the association between baseline RAFH and each of the following baseline characteristics separately (fixed-effects): age, sex, BMI, history of smoking, presence of CVD, baseline focality of GA lesions (unifocal vs. multifocal), baseline hyperautofluorescence pattern (group 1: “None,” “Focal” vs. group 2: “Banded,” “Patchy,” “Diffuse”), baseline perimeter, and baseline square root-transformed GA area. We then chose all characteristics that were statistically significantly associated with baseline RAFH (*P* < 0.05) in the univariate models and entered them in a multivariable mixed-effects model that was constructed similarly. In the analysis investigating the impact of metformin on RAFH growth, we included patients with varying levels of follow-up visits up until 18 months. At that time, metformin administration was discontinued per the METforMIN trial protocol. We modeled RAFH as a function of fixed effects of time, treatment arm, and the interaction between the 2, with nested random intercepts for eye (to account for repeated measures in the same eye) and crossed random intercept for participant (to account for the correlation of eyes from the same person) as well as a random slope for eye across study visits.^34^ The model assessing the longitudinal change of RAFH over 24 months was constructed similarly except for removing predictor variables of the treatment arm and the interaction between time and treatment arm.

## Results

### Participant Characteristics and Intergrader Reproducibility of RAFH

**Figure 2:**
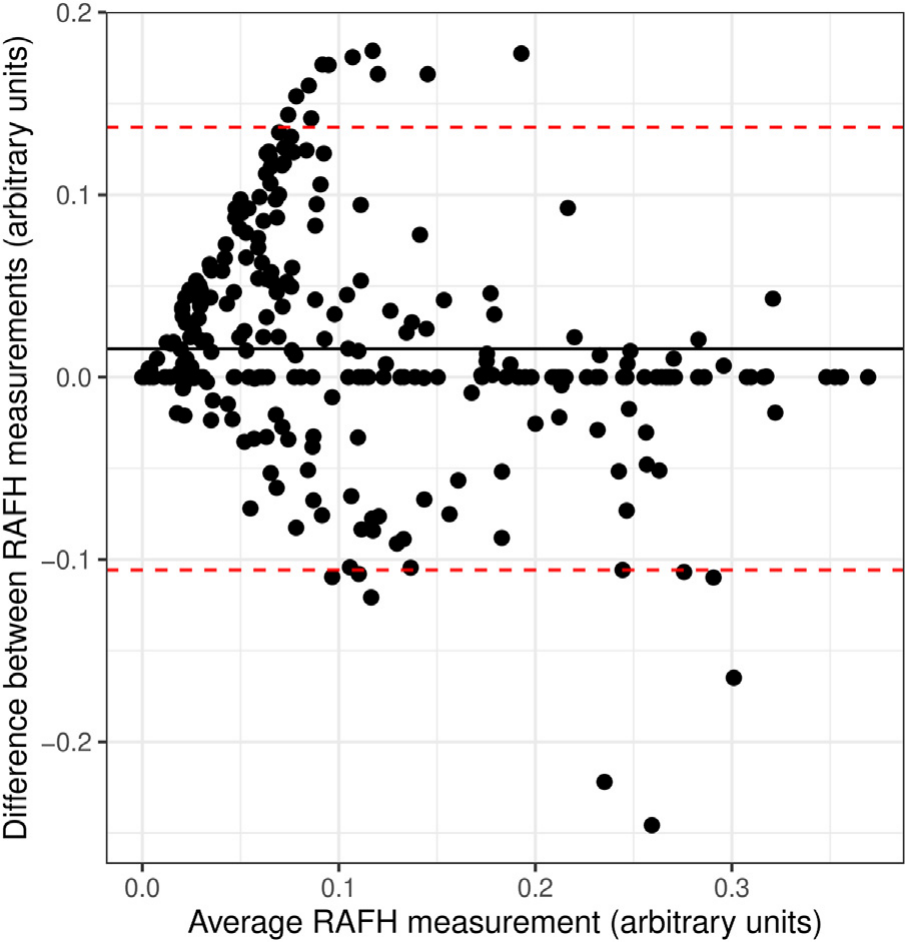
Bland–Altman plot showing comparisons between the RAFH measurements of 2 graders (N = 282 images). Overall, RAFH had a mean difference of 0.016 (the solid line) and 95% limits of agreement of −0.106 to 0.137 (dashed lines), with an intraclass correlation coefficient of 0.78. RAFH = rim area focal hyperautofluorescence.

Seventy-one eyes (34 metformin; 37 observation) from 44 participants (21 metformin; 23 observation) were included in the final analysis. Two hundred eighty-two total FAF images (136 metformin; 146 observation), with each image belonging to a single visit, were graded separately by the 2 graders. Further baseline characteristics of participants can be seen in Table 1. The intraclass correlation coefficient between the 2 graders after corrections was 0.78 (95% confidence interval, 0.72–0.83) with a mean difference of 0.016, and Bland–Altman limits of agreement between −0.106 and 0.137 (Fig 2). Sixteen of the 282 (5.7%) images had RAFH grading outside the limits of agreement, requiring a discussion and mutual agreement between the 2 graders.

**Table 1:** Baseline Clinical and Imaging Characteristics of Study Participants

Characteristic	Metformin	Observation	*P* Value∗
	N = 34 Eyes; 21 Participants	N = 37 Eyes; 23 Participants	

Age (yrs), median (IQR)	74 (68–83)	78 (75–81)	0.12
Male sex, n (%)	14 (41)	10 (27)	0.21
FAF phenotype classification, n (%)			0.45
“None” and “Focal”	22 (65)	27 (73)	
“Banded,” “Patchy,” and “Diffuse”	12 (35)	10 (27)	
Foveal involvement, n (%)	29 (85)	32 (86)	>0.99
Longest follow-up interval (yrs), median (IQR)	1.99 (1.52–2.05)	1.63 (1.45–2.00)	0.25

FAF = fundus autofluorescence; IQR = interquartile range.

Wilcox rank sum test is used for all continuous variables; chi-square test of independence is used for expected cell count >5, whereas Fisher exact test is used for expected cell count <5.

n (%) refers to number of eyes.

∗ Wilcoxon rank sum test; Pearson chi-squared test; Fisher exact test.

### Baseline RAFH Was Associated with Baseline GA Area

Baseline RAFH was positively associated with the baseline square root of GA area (*P* < 0.001) and baseline GA perimeter (*P* < 0.01) based on the univariate analysis, but not with age, sex, BMI, focality of baseline GA lesions, FAF pattern, as well as history of smoking or CVD (Table 2). In the multivariate model, only the baseline square root of GA area was significantly associated with baseline RAFH (*P* < 0.001).

**Table 2:** Cross-Sectional Association of Baseline RAFH with Clinical and Imaging Characteristics

Baseline Factor	Category	Number of Eyes	Univariate model∗		Multivariate model†	
			Coefficient Estimate (95% CI), Arb. units	*P* Value‡	Coefficient Estimate (95% CI), Arb. units	*P* Value‡
Lesion number	-	71	0.002 (−0.006, 0.01)	0.64		
Square root of GA area, mm	-	71	0.053 (0.036, 0.07)	**<0.001**	0.065 (0.039, 0.09)	**<0.001**
GA perimeter, mm	-	71	0.002 (0.001, 0.004)	**0.003**	−0.001 (−0.003,0.00)	0.22
BMI	-	63	−0.003 (−0.008, 0.002)	0.21		
Age, yrs	-	71	0.00 (−0.003, 0.002)	0.79		
Sex	Male	23	−0.018 (−0.069, 0.033)	0.48		
	Female	48	Reference	-		
CVD history	Yes	38	0.001 (−0.048, 0.049)	0.97		
	No	33	Reference	-		
Smoking history	Yes	26	0.016 (−0.034, 0.065)	0.53		
	No	45	Reference	-		
FAF pattern	“Banded,” “Patchy,” and “Diffuse”	23	0.005 (−0.035, 0.045)	0.79		
	"None" and "focal"	48	Reference	-		
GA foveal involvement	Yes	63	−0.006 (−0.063, 0.051)	0.83		
	No	8	Reference			
GA focality	Multifocal	47	−0.015 (−0.054, 0.025)	0.47		
	Unifocal	24	Reference	-		

BMI = body mass index; CI = confidence interval; CVD = cardiovascular disease; FAF = fundus autofluorescence; GA = geographic atrophy; RAFH = rim area focal hyperautofluorescence.

∗ Factors were entered one-by-one separately in the univariate model.

† Only factors with univariate *P* < 0.05 were included in the multivariate model.

‡ Values in bold denote statistical significance at *P* < 0.05.

### Increased Baseline RAFH Was Associated with Faster GA Progression

**Figure 3:**
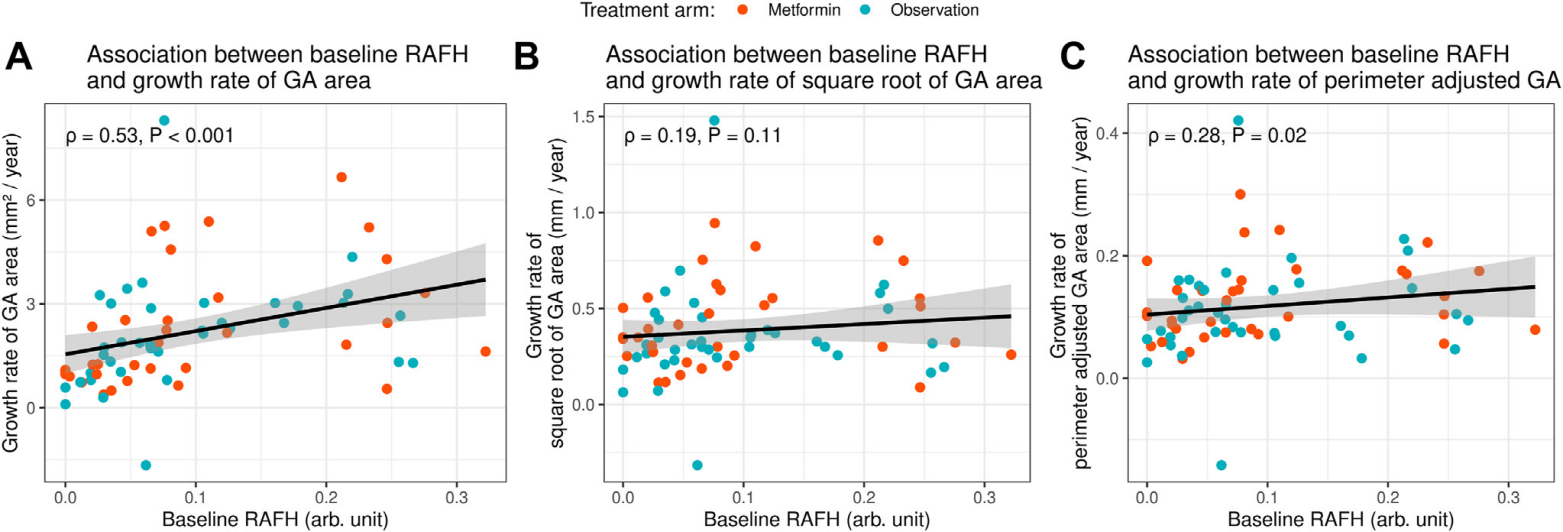
Spearman plots with correlation coefficients (ρ) and *P* values showing the association between baseline RAFH and **A**, growth rate of GA area (mm^2^/year); **B**, growth rate of square root-transformed GA area (mm/year); and **C**, perimeter-adjusted GA growth rate (mm/year). The growth rate of GA area (mm^2^/year) was positively associated with baseline RAFH (ρ = 0.53 and *P* < 0.001). The association remained positive but became weaker in square root-transformed GA area growth (B, ρ = 0.19 and *P* = 0.11) and perimeter-adjusted GA growth rate (C, ρ = 0.28 and *P* = 0.02). The gray shaded area surrounding the line represents standard error. N = 71 eyes. arb. unit = arbitrary units; GA = geographic atrophy; RAFH = rim area focal hyperautofluorescence.

The growth rate of GA area (mm^2^/year) was positively associated with baseline RAFH (Spearman’s ρ = 0.53 and *P* < 0.001, Fig 3A). The association remained positive but became weaker in square root-transformed GA area growth (ρ = 0.19 and *P* = 0.11, Fig 3B) and perimeter-adjusted GA growth rate (ρ = 0.28 and *P* = 0.02, Fig 3C).

**Figure 4:**
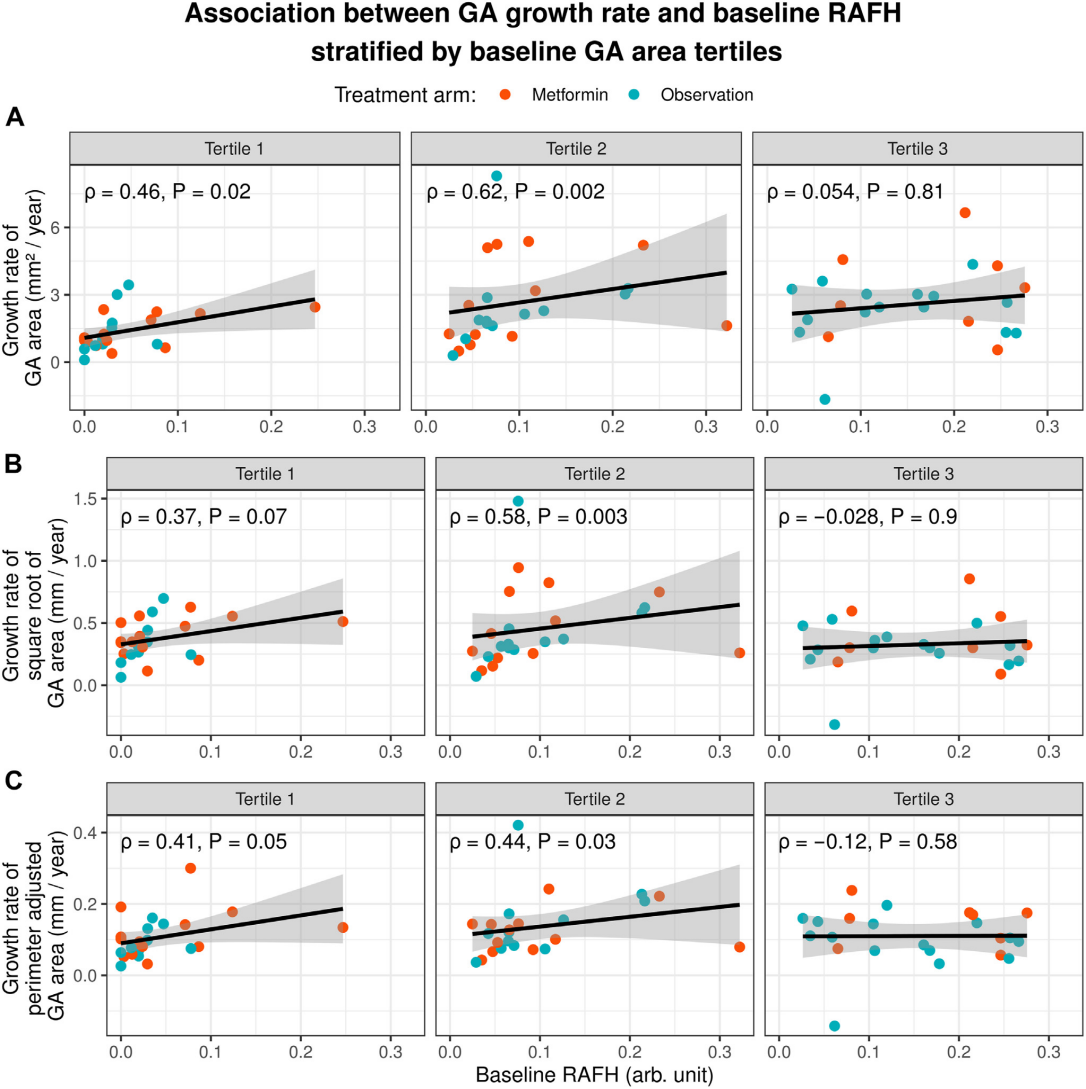
Spearman plots with correlation coefficients (ρ) and *P* values showing the association between baseline RAFH and GA growth rate stratified by baseline GA area tertiles. Tertiles are arranged in increasing order of baseline GA area. **A,** Growth rate of GA area in (mm^2^/year). **B,** Square root-transformed GA area growth in (mm/year). **C,** Perimeter-adjusted GA area growth rate in (mm/year). In all 3 panels (**A**, **B**, **C**), GA growth rate was positively associated with baseline RAFH in tertiles 1 and 2, but the relationship was not statistically significant in tertile 3, suggesting that RAFH may predict GA growth more in the earlier stage than in the later stage of the disease course. The gray shaded area surrounding the line represents standard error. N = 71 eyes in each panel. arb. unit = arbitrary units; GA = geographic atrophy; RAFH = rim area focal hyperautofluorescence.

After we stratified eyes into 3 tertiles based on baseline GA area, the association between baseline RAFH and GA growth rate remained positive and statistically significant in the first and second tertile for GA area growth (mm^2^/year) and perimeter adjusted GA growth (in mm/year), and in the second tertile for square root-transformed GA growth rate (in mm/year) (Fig 4). Geographic atrophy growth rate was not significantly associated with baseline RAFH in the third GA size tertile using any of the 3 GA growth rate measurements.

### RAFH Increases over Time

**Figure 5:**
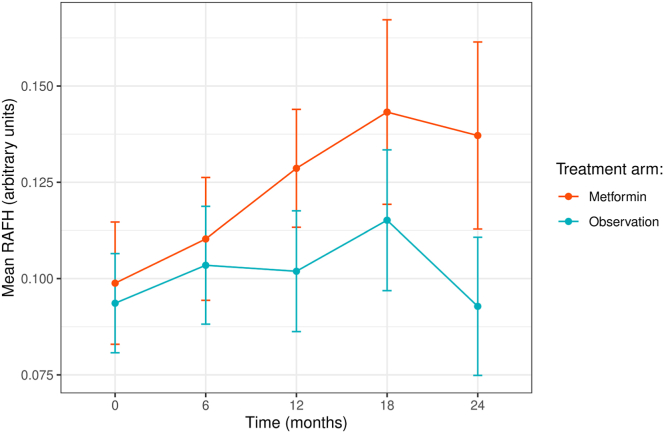
Mean RAFH over time of participants in each arm. Vertical bars represent standard error. The annualized change rate ± standard error of mean RAFH in the metformin group was 0.023 ± 0.009 units/year (34 eyes from 21 participants) and 0.016 ± 0.008 units/year in the observation group (37 eyes from 23 participants). Oral metformin did not significantly impact the change rate of RAFH over 18 months (*P* = 0.29). RAFH = rim area focal hyperautofluorescence.

In the combined metformin and observation group, RAFH increased slightly but significantly at 0.020 ± 0.006 units/year (*P* = 0.002) over 24 months. The annualized change rate of mean RAFH was 0.023 ± 0.009 units/year in the metformin group (34 eyes from 21 participants) and 0.016 ± 0.008 units/year in the observation group (37 eyes from 23 participants) (Fig 5). Oral metformin did not significantly affect the change in RAFH over 18 months (*P* = 0.29). After removing 5 eyes from 3 participants who were <75% adherent to oral metformin, the results did not change significantly (*P* = 0.37).

## Discussion

To our knowledge, this is the first study to investigate the association of baseline RAFH and GA progression after accounting for baseline GA area and perimeter. We found that RAFH was significantly higher in eyes with larger GA size. A larger baseline RAFH was significantly associated with faster GA area progression (in mm^2^/year) (ρ = 0.53 and *P* < 0.001). The association remained positive but became weaker after we used the growth rate of square root-transformed GA area (ρ = 0.19 and *P* = 0.11) and perimeter-adjusted growth rate (ρ = 0.28 and *P* = 0.02) in mm/year, suggesting that the previously reported association between baseline RAFH and GA area growth rate in mm^2^/year may be partly confounded by baseline GA area and perimeter.^24^, ^25^, ^26^ Although it is well known that the square root transformation (mm/year) is preferred to track GA growth over the area growth in mm^2^/year,^28^^,^^45^ recent clinical trials still reported changes in GA size in mm^2^.^3^^,^^4^ Our study further found RAFH to increase slightly but significantly over time, and oral metformin did not significantly affect the change rate of RAFH.

Our overall Spearman’s correlation coefficient of 0.53 (*P* < 0.001) between RAFH and GA area growth rate (mm^2^/year) is comparable to previously reported values of 0.49^24^ and 0.60.^26^ When using square root-transformed or perimeter-adjusted GA growth rate, the reduced correlation strength suggests that the observed association between baseline RAFH and GA area growth rate may be partly confounded by baseline GA area and perimeter. This result highlights the advantage of either of the 2 methods—square root-transformed and perimeter-adjusted—when calculating GA growth rate as an endpoint,^9^^,^^28^^,^^30^^,^^45^^,^^46^ as opposed to the traditional approach in mm^2^/year. In addition to adjusting for baseline GA area, few studies have shown that the perimeter-adjusted GA growth also adjusts for additional features, such as the number of GA lesions and their circularity index.^30^^,^^47^ Interestingly, we found a moderate positive correlation between RAFH and square root-transformed as well as perimeter-adjusted GA growth rate in the first 2 baseline GA area tertiles but not in the third baseline GA area tertile (Fig 4), suggesting that baseline RAFH may have a better prognostic value in small and medium-sized GA (under 8.0 mm^2^) than in large GA (above 8.2 mm^2^).^48^^,^^49^ The exact reason for this phenomenon is unclear. One explanation is that GA lesions of tertiles 1 and 2 may be at different stages of the natural history of the disease and, therefore, display different GA growth rates.^48^, ^49^, ^50^, ^51^ This is supported by studies that found a sigmoidal pattern of GA growth rate to be more representative of the natural course of atrophic lesions.^49^^,^^52^ Smaller lesions may also vary in topographical distribution more than large, coalesced lesions, which may result in distinct growth patterns since locations farther away from the fovea tend to exhibit faster rates.^50^ The pathological underpinning for chronological and topographical variations in GA growth rate is also not fully understood.^51^ However, some studies have implicated that differences in macular pigment distribution, higher susceptibility of rods to atrophy progression, and the presence of genetic variants of pathological significance play an important role in explaining this variation.^8^^,^^50^^,^^51^^,^^53^^,^^54^ With the advent of complement inhibitors to slow the rate of GA progression,^55^ the predictive value of RAFH may help clinicians identify patients who could benefit the most from such pharmacological therapy. The correlation between RAFH and the extent of photoreceptor loss on OCT in the junctional area may also offer insights into GA progression dynamics^56^ and should be the subject of future studies as deep learning algorithms required for such analysis become more readily available.^57^

The hyperautofluorescent signals surrounding GA lesions were initially thought to represent lipofuscin-accumulated RPE cells at a higher risk of dying.^17^^,^^18^ However, more recent studies have associated these signals with morphological changes such as vertical stacking, migration, or redistribution of RPE cells.^58^, ^59^, ^60^, ^61^ Some histological studies characterize the border of atrophy as an area undergoing RPE cell apoptosis and transdifferentiation, thus contributing to spatio-morphological alterations in the RPE layer.^60^^,^^62^ As such, the migration of RPE cells may correspond with the local movement of signals on FAF or with hyperreflective foci on OCT, as some have suggested.^62^^,^^63^ In this context, the association between greater RAFH and faster GA progression in tertiles 1 and 2 may indicate an overall state of high RPE stress and disease activity, though it is unknown why this relationship is restricted to small and medium GA (under 8.0 mm^2^). Although histologic autofluorescence per RPE cell is reported to decrease with increasing RPE cell dysmorphia,^63^, ^64^, ^65^ high interindividual autofluorescence variation in normal eyes,^66^ the effect of age and retinal location,^62^^,^^63^^,^^66^ aggregation of RPE cells or pigment granules,^62^^,^^64^^,^^65^ and the phenomena of RPE shedding and degranulation,^60^^,^^63^^,^^64^^,^^67^ may contribute substantial variation and thus, pose a significant challenge in elucidating the relationship between autofluorescence and RPE pathology. Future studies may focus on corresponding hyperautofluorescence signals on FAF with hyperreflective foci on OCT in the junctional area of GA, which may further illuminate the role of each biomarker in predicting GA progression.

Another potential biomarker previously reported is an increase in the thickness of the sub-RPE complex found near the atrophic border, which may correspond to RPE distress and structural alterations.^60^^,^^68^ This is also known as basal laminar deposits. Some reports have suggested the pronounced thickening of basal laminar deposits in the “diffuse-trickling” FAF phenotype to potentially explain extraordinarily fast GA progression.^69^, ^70^, ^71^ Since these deposits may contain aggregates of autofluorescent granules,^72^ they can potentially alter the autofluorescence seen on FAF imaging.^60^ Future studies may aim to correlate the thickness of basal laminar deposits in different FAF phenotypes with their respective GA growth rates using OCT.

In this study, we also investigated whether baseline RAFH is associated with risk factors previously reported with the progression of GA, including clinical characteristics (age,^8^ sex,^73^ BMI,^74^ smoking history,^74^ and history of CVD^73^) as well as morphological GA factors (baseline lesion focality,^8^^,^^30^ baseline lesion number,^30^^,^^50^ hyperautofluorescence pattern,^35^ baseline GA perimeter,^30^^,^^75^ and baseline square root-transformed GA area^29^). The only statistically significant association of baseline RAFH with baseline square root-transformed GA area reinforces that bigger GA lesions may be at a later stage in their natural course of the disease than the smaller ones, hence showing different extents of RAFH at their border.^48^^,^^76^ The lack of association of RAFH with clinical risk factors associated with the incidence and progression of GA may be due to our small sample size. We also recognize that this analysis did not encompass all GA morphological features associated with GA progression. One such example is GA lesion diameter.^77^ However, we did include GA perimeter in our analysis, which is closely related to GA lesion diameter. Correlating clinical characteristics of patients with RAFH in larger studies may help us further our understanding of the pathological role of hyperautofluorescence signals in GA progression.

Our last goal of this study was to assess the change in RAFH over time and investigate the impact of oral metformin on minimizing RAFH growth. The limited effect of oral metformin on influencing RAFH’s growth may be attributed to a lack of statistical power to detect notable differences, insufficient follow-up time, inappropriate dosing, and the possibility of a lack of biological effect of the medication on this disease process.^34^ Thus far, no studies have looked at the evolution of RAFH longitudinally. The temporal changes in RAFH may offer insights into the pathogenesis of GA growth. For example, after inducing diffuse outer retinal injury using intravenous injection of sodium iodate in a rat model, Pankova et al tracked the formation of hyperautofluorescent regions. They found hyperautofluorescent patterns to be spatially dynamic. Over 2 months, hyperautofluorescent areas percolated inwards from the peripheral retina and morphed from distinct bright perilesional areas to diffuse granular in appearance.^31^ Similarly, using subretinal injections of sodium iodate in rats, another study generated distinct regions of GA, which showed multilayered stacking of RPE at the GA border and anterior retinal migration of detached RPE cells on histology.^78^ Changes in the intensity of autofluorescence and its migration are essential features, as they may determine the amount of hyperautofluorescence within the 450-μm border of atrophy. The mobilization of RPE cells at the border of a healthy retina and GA was also suggested by Biarnés et al, who proposed that junctional hyperautofluorescent regions may be the result of GA enlargement rather than the cause of it via an unknown underlying process.^76^^,^^79^ Some studies have implied migrating RPE cells to be undergoing transdifferentiation from epithelial to mesenchymal origin based on loss of immunoreactivity to retinoid markers^80^ and changes in emission spectra.^81^ However, others have classified these migrating cells as macrophages with engulfed melanofuscin granules.^82^ Thus, the increase in RAFH over time observed in our study may be a manifestation of the slow mobilization of RPE cells, which in turn may adopt different morphologies, such as vertical stacking. Alternatively, it may be a marker of the high burden of melanophages secondary to increased RPE stress and dysmorphia. How this mobilization relates to GA growth and underlying pathogenesis warrants further investigation. However, it is imperative to note that variation in camera position between serial visits for the same eye may influence the quantification of autofluorescence at the border of GA,^83^ which can potentially confound the measurement of RAFH over time. Instruments with an internal autofluorescence reference, such as quantitative autofluorescence, may be used to align specific locations in quantitative autofluorescence images with OCT scans to elucidate the relationship between different biomarkers further.^84^^,^^85^

This study has several limitations. First, our intergrader intraclass correlation coefficient of 0.78 is acceptable but not excellent, which may have influenced our ability to establish the association between RAFH and GA growth. However, our mean difference (lower 95%, upper 95%) between the 2 graders was 0.016 (−0.106, 0.137), comparable to Allingham et al’s mean differences, which ranged between −0.005 and 0.017.^24^ We believe that variations in the lens and media opacity,^86^ patient positioning,^24^^,^^25^^,^^83^ eye movements,^24^^,^^83^ and camera alignment^24^^,^^83^ are some factors that are bound to impact people’s grading in different ways. To mitigate this impact, we used the average RAFH of the 2 graders. The METforMIN trial also did not document the lens status of the participants, which may also confound FAF intensity.

Second, our sample size could be bigger, which likely limited the power of some of our analyses. Furthermore, in the METforMIN trial population, participants either dropped from the study or missed interval visits between the baseline visit and the prespecified follow-up interval of 24 months, which may have introduced bias.

In conclusion, increased baseline RAFH was associated with faster GA area progression. The association remained positive but weakened after adjusting for baseline GA area or perimeter in the entire cohort. However, tertile analysis showed that baseline RAFH had a stronger prognostic value for GA growth rate among eyes with small and medium GA lesions (less than 8.0 mm^2^) than large GA (greater than 8.2 mm^2^). This finding could help designate pharmacological treatments for patients with the highest risk of GA progression. Rim area focal hyperautofluorescence increased significantly over time, and oral metformin did not significantly affect the growth rate of RAFH. Future studies may consider using baseline RAFH as a prognostic factor for eyes with small and medium GA.
