# Supplementary material for: Association of Hyperautofluorescence Signals with Geographic Atrophy Progression in the METformin for the MINimization of Geographic Atrophy Progression Trial
Source: Ophthalmol Sci. 2024 Sep 12;5(1):100620. doi: 10.1016/j.xops.2024.100620 (PMC11585696; doi:10.1016/j.xops.2024.100620)
Supplement: Supplemental Methods [file mmc1.pdf]

Supplemental Methods. Geographic atrophy grading and fundus autofluorescence phenotyping process.

All geographic atrophy (GA) grading in this manuscript was adopted unchanged from the METforMIN trial, during which a total of 353 fundus autofluorescence (FAF) images were graded by six independent graders for the total area of GA lesions, GA lesion focality (unifocal vs. multifocal), and foveal center involvement in the GA. Each image was graded by two independent graders masked to treatment allocation. The first 185 collected FAF images were graded by two professional graders at the University of California, Davis (UCD) reading center. The lack of funding for the study necessitated the remaining 168 images were graded by four trained graders, including three medical students (JS, AT, and NC) and one ophthalmology resident physician (LS), at the University of California, San Francisco (UCSF), using the same grading protocol. To investigate the intergrader reproducibility between UCSF and UCD, we randomly selected 24 images (six images per GA area quartile) that were graded by UCD and asked each UCSF grader to grade the 24 images. The intraclass correlation coefficient of the GA area between each UCSF grader and UCD grader ranged from 0.94 to 0.98. Then, we randomly divided the FAF images not graded by the UCD reading center into image sets A and B. JS and AT independently graded each image in image set A, and LS and NC independently graded each image in image set B. The graders also assessed optical coherence tomography images for GA involvement of the foveal center point. Images graded by the three medical students were reviewed by two expert graders (ophthalmology resident physicians) to ensure accuracy. The expert graders corrected GA tracing errors estimated to affect at least 10% of the total GA area measurement. The two expert graders also resolved disagreements between graders in GA presence, lesion focality, FAF autofluorescence pattern, and foveal center point involvement through open arbitration. The total GA area for each FAF image was calculated as the mean GA area between two graders.

The baseline FAF patterns for 71 eyes in this study were graded in the same manner as above by the same graders at UCD and UCSF. The patterns were classified into two groups, group 1: “None” and “Focal” and group 2: “Banded,” “Patchy,” and “Diffuse.” Upon the reviewer's request, these patterns were re-graded by AT and AD to include the “Diffuse Trickling” phenotype in addition to “None,” “Focal,” “Banded,” “Patchy,” and “Other Diffuse.” However, the Kappa (κ) statistic of 0.08, interpreted as “slight” agreement between the AT and AD, was not deemed precise enough to include these results in the manuscript. As such, the authors opted to continue to use the previous classification of 2 groups.
